# The Relationship Between Health Literacy and Mental Health Attitudes and Beliefs

**DOI:** 10.3928/24748307-20221018-01

**Published:** 2022-10

**Authors:** Sasha A. Fleary, Patrece L. Joseph, Carolina Gonçalves, Jessica Somogie, Jessica Angeles

## Abstract

**Background:**

Mental health first aid programs show promise in reducing stigma and increasing help-seeking. However, the success of these and other mental health interventions are likely affected by health literacy. Yet, health literacy is understudied in the mental health literature and rarely considered in mental health interventions.

**Objective:**

This study explored the relationship between health literacy and mental health stigma, aversion to mental health help-seeking, and willingness to interact with individuals with mental illnesses.

**Methods:**

Adults (*N* = 601, mean age = 45.64) completed online surveys of their health literacy and mental health attitudes and beliefs. Hierarchal linear regression models were estimated to examine the relationship between health literacy and mental health attitudes and beliefs. Path models were estimated to determine if stigma mediated the relationship between health literacy and (1) aversion to help-seeking and (2) willingness to interact with individuals with mental illnesses.

**Key Results:**

Adults with higher functional and communicative health literacy had lower mental health stigma and aversion to mental health help-seeking. Adults with higher communicative health literacy and empowerment were more willing to interact with individuals with mental illnesses. Mental health stigma partially mediated the relationships between communicative health literacy and aversion to mental health help-seeking and willingness to interact with individuals with mental illnesses. Mental health stigma fully mediated the relationships between functional health literacy and aversion to mental health help-seeking and willingness to interact with individuals with mental illnesses.

**Conclusions:**

Results support including health literacy in mental health interventions and reiterate addressing stigma in community and clinical settings. [***HLRP: Health Literacy Research and Practice*. 2022;6(4):e270–e279.**]

**Plain Language Summary:**

Many adults with mental health problems do not get help because of negative beliefs about mental health. We found that adults with more skills for accessing, understanding, and using health information had fewer negative opinions and were more willing to interact with others with mental health problems. Improving those skills may reduce negative opinions about mental health and seeking help.

In the United States, about 20% of adults have mental health (MH) problems and less than 45% receive MH services (National Institute of Mental Health, 2022). In addition to challenges accessing treatment (Rastogi et al., 2012; Rhodes et al., 2009; Salaheddin & Mason, 2016; Walker et al., 2015) and lower awareness of MH problems (Rüsch et al., 2011), MH stigma (Clement et al., 2015; Henderson et al., 2013; Salaheddin & Mason, 2016; Schnyder et al., 2017) is a major barrier to help-seeking. MH stigma includes negative stereotyping, labeling, separation, discrimination, and disempowerment of individuals with mental illnesses (Link & Phelan, 2001). According to Ahmedani (2011), MH stigma includes social stigma (viewing individuals with stigmatized conditions as inferior), self-stigma (internalizing negative stereotypes associated with one's condition) and health professional stigma (treatment of patients affected by providers' stigmatizing attitudes).

Stigma may be reduced by improving MH literacy (Cheng et al., 2018; Sickel et al., 2019), knowledge and beliefs about mental disorders that assist with treatment, management, and prevention. Several aspects of MH literacy are associated with help-seeking and stigma. For example, the ability to recognize MH disorders is linked to positive help-seeking attitudes (Cheng et al., 2018), whereas better knowledge about mental illness is associated with self-identification of mental illness (Schomerus et al., 2019) and attitudes supporting community care (Rüsch et al., 2014). MH first aid training programs teach community members to identify, understand, respond to, and support individuals with mental illnesses and have successfully reduced stigma (Burns et al., 2017; Hadlaczky et al., 2014; Jorm, 2012; Morgan et al., 2018).

A gap in the MH help-seeking and stigma literatures is the potentially protective role of health literacy (HL). Ahmedani (2011) argues that social stigma is deeply rooted in society. Because individuals develop in an ecological context (Bronfenbrenner & Morris, 2007) in which stigma is embedded, stigma-related beliefs and attitudes may be socialized via these contexts. The extent to which these attitudes persist, and impact MH help-seeking may depend on HL. Individual-level HL is how one accesses, understands, evaluates, and uses health information in their decision-making for disease treatment and prevention and health promotion (Sørensen et al., 2012). Individuals with higher HL may be better equipped to critically analyze negative attitudes about MH and less likely to ascribe to stigmatization. Although several interventions address MH social stigma and self-stigma (Hanisch et al., 2016; Yanos et al., 2015), none have considered HL.

Three core qualities of HL include functional (reading, writing, and numeracy), interactive/communicative (use of health knowledge to engage with the environment/others about health), and critical HL (self and community advocacy) per Nutbeam (2008) and Sørensen et al. (2012). HL is positively associated with preventive health behaviors (Aaby et al., 2017), help-seeking for chronic conditions and communication with medical providers (White et al., 2016; Yin et al., 2012). These skills likely influence MH help-seeking, beliefs, and attitudes. The success of MH first aid programs and similar interventions are likely affected by participants' HL as the uptake of information shared and engagement in actions suggested by these programs require a combination of functional, communicative, and critical HL skills.

This study explored the relationship between HL and MH social stigma, help-seeking attitudes, and willingness to interact with individuals with mental illnesses (willingness to interact). Although willingness to interact may be conceptualized as stigma, it is also an outcome of social stigma and treated as such in our study. We hypothesized that individuals with higher HL would have lower stigma, lower aversion to help-seeking, and higher willingness to interact than those with lower HL. We also hypothesized that MH stigma would mediate the relationship between (1) HL and aversion to help-seeking and (2) HL and willingness to interact.

## Method

### Participants and Procedures

Study procedures were approved by the Tufts University Social, Behavioral, and Educational Research Institutional Review Board (Protocol # 1709022). Data were collected from a Qualtrics survey panel (*n* = 500) and through social media recruitment (*n* = 101) from March 2018 to October 2018. For the Qualtrics survey panel, participants were recruited from a third-party panel that randomly selects participants based on researchers' needs. Researchers requested the panel sample match the demographic characteristics of the U.S., with an oversampling for racial and ethnic minority groups to reduce variances of key statistics for these groups. Panel staff sent a survey link to eligible adults in their panel. For social media recruitment, the study team posted a link to the Qualtrics survey on social media inviting adults age 18 years and older to participate in the study and share the survey link. After accessing the survey link, participants provided informed consent, then completed the survey before viewing a debriefing page with MH resources. The survey took about 30 minutes to complete. Attention-check questions were embedded throughout the survey. The Qualtrics survey panel sample was compensated with incentives set by Qualtrics (e.g., cash, redeemable points). The social media sample received a $15 e-gift card.

### Measures

***Demographic covariates***. Participants self-reported their age (years), gender (male, female, transgender, male-to-female transgender, female-to-male transgender, gender non-conforming, other), and education level (less than high school, high school, some college, Associate's, Bachelor's, Graduate degree). Participants indicated their race and ethnicity: Hispanic/Latino/a/e or Spanish origin; Black or African American; Asian; Native American or Alaska Native; Native Hawaiian or Other Pacific Islander; White. The social media sample could select multiple races. The Qualtrics sample could only select one race due to logistics determining quotas. Native American or Alaskan Native, Native Hawaiian or Other Pacific Islander and Multiracial were collapsed into a single group due to small sample sizes. Responses were re-coded where indicated (e.g., functional HL questions 1 and 3). Participants also responded to two questions about if they or family members had a known diagnosis of MH problems. A *yes* response to either question was coded as yes for the self/family history of mental illness variable. All questions included a prefer not to answer option which was set to missing for the analyses.

***HL***. The 13-item All Aspects of Health Literacy Scale (Chinn & McCarthy, 2013) measures functional, communicative, and critical HL, and empowerment. It includes three functional HL (e.g., How often do you need help to fill in official documents?); three communicative HL (e.g., When you talk to a doctor or nurse, do you ask the questions you need to ask?); four critical HL (e.g., Are you someone who likes to find out lots of different information about your health?); and three empowerment questions (e.g., Within the last 12 months have you taken action to do something about a health issue?). Options for the functional, communicative, and critical HL questions were sometimes, often, or always. Options for the empowerment questions varied. Participants' subscale scores were summed; higher scores represented higher HL. Cronbach reliability alpha for the measure was 0.75; alpha >.70 is acceptable (Cortina, 1993).

***MH attitudes.*** MH attitudes were assessed using 12 items from the 35-item Mental Health Literacy Scale (O'Connor & Casey, 2015), a measure of MH knowledge and attitudes toward help-seeking. Three factors emerged from an exploratory factor analysis (stigma, willingness to interact, aversion to MH help-seeking). A confirmatory factor analysis supported the 3-class solution. Six MH social stigma questions (Cronbach's alpha = 0.86) assessed participants' attitudes toward individuals with mental illness (e.g., People with a mental illness are dangerous). Aversion to MH help-seeking included three questions (Cronbach's alpha = 0.70) about participants' beliefs about not disclosing a mental illness to others and not seeking treatment (e.g., Seeing a MH professional means you are not strong enough to manage your own difficulties). Response options for these 9 questions were on a 5-point scale ranging from strongly disagree to strongly agree. Three items (Cronbach's alpha = 0.85) assessed individuals' willingness to interact with those with mental illnesses (e.g., How willing would you be to make friends with someone with a mental illness?). Response options were on a 5-point scale ranging from *definitely unwilling* to *definitely willing*. Mean (M) scores were computed for all subscales with higher scores indicating higher MH stigma, aversion to MH help-seeking, and willingness to interact.

### Statistical Analyses

**Figure 1.**
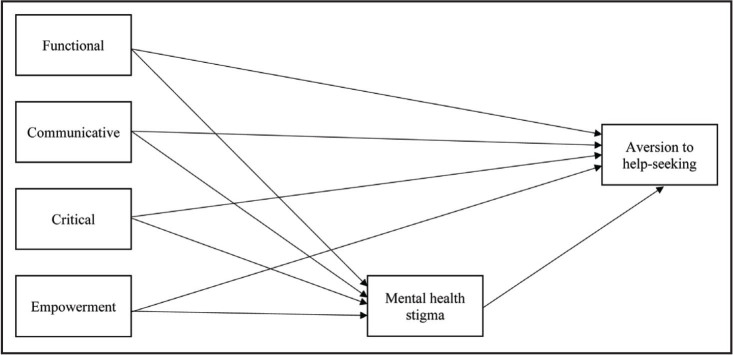
Path model of mental health stigma mediating the relationship between health literacy and aversion to mental health help-seeking. Note. The path model for Model 2 is the same except willingness to interact with individuals with mental illness is the outcome variable.

Analyses were conducted in SPSS 27 and Mplus Version 7 (Muthén & Muthén, 2012). Descriptive statistics were computed. Hierarchical linear regressions were estimated (demographics in step 1, HL in step 2) to determine the relationship between HL and the MH variables. Two path models (**Figure 1** illustrates the path model) were estimated in Mplus using maximum likelihood estimation to examine whether MH stigma mediated the relationship between HL and (1) aversion to MH help-seeking and (2) willingness to interact. All estimated models included demographic covariates that were significantly associated with any of the MH variables. Direct and indirect effects were examined to assess MH stigma as a mediator between HL and the outcomes. The estimated models were “just-identified”: the number of variances and covariances were equal to the number of estimated parameters resulting in 0 degrees of freedom. Interpreting just-identified models is appropriate depending on the models and research questions (Kenny, 2011; UCLA: Statistical Consulting Group, n.d.). Beta weights and variances are used to interpret just-identified models rather than global fit statistics (UCLA: Statistical Consulting Group, n.d.).

## Results

Demographic characteristics are presented in **Table 1** and **Table 2**. The sample included 601 adults (Mean *[M]* age = 45.64; about 62% female, about 22% Hispanic/Latino/a/e). The largest racial group was White (about 43%), about 41% of adults had a Bachelor's or Graduate degree, and about 36% reported a self and/or family history of mental illness diagnosis. The average HL scores were as follows: functional (*M* = 8.02, standard deviation [*SD*] = 1.20), communicative (*M* = 8.17, *SD* = 1.36), critical (*M* = 9.39, *SD* = 2.00), and empowerment (*M* = 4.52, *SD* = 0.81). MH stigma was positively correlated with aversion to help-seeking (*r* = 0.62, *p* < .001). Willingness to interact (*r* = −0.33, *p* < .001) was negatively correlated with MH stigma and aversion to help-seeking (r = −0.29, *p* < .001). Only significant regression and path model findings are discussed below.

**Table 1:**
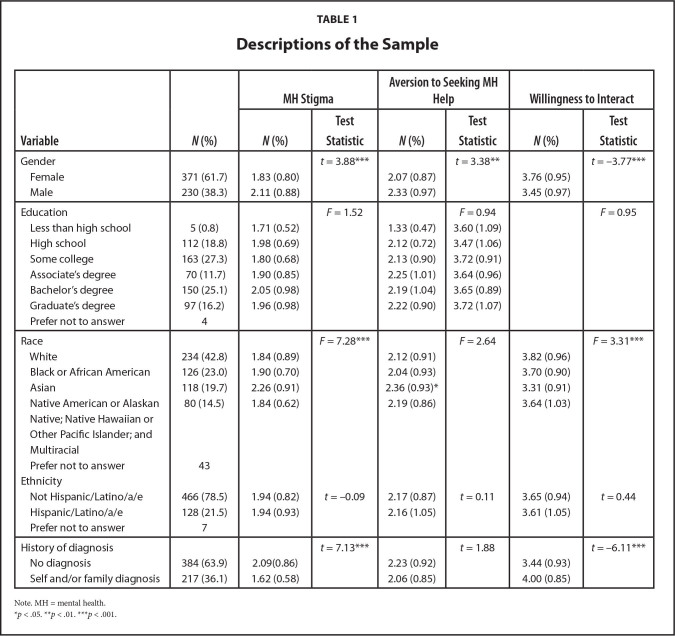
Descriptions of the Sample

**Variable**	***N* (%)**	**MH Stigma**		**Aversion to Seeking MH Help**		**Willingness to Interact**	
		
		***N* (%)**	**Test Statistic**	***N* (%)**	**Test Statistic**	***N* (%)**	**Test Statistic**

Gender			*t* = 3.88^***^		*t* = 3.38^**^		*t* = −3.77^***^
Female	371 (61.7)	1.83 (0.80)		2.07 (0.87)		3.76 (0.95)	
Male	230 (38.3)	2.11 (0.88)		2.33 (0.97)		3.45 (0.97)	

Education			*F* = 1.52		*F* = 0.94		*F* = 0.95
Less than high school	5 (0.8)	1.71 (0.52)		1.33 (0.47)	3.60 (1.09)		
High school	112 (18.8)	1.98 (0.69)		2.12 (0.72)	3.47 (1.06)		
Some college	163 (27.3)	1.80 (0.68)		2.13 (0.90)	3.72 (0.91)		
Associate's degree	70 (11.7)	1.90 (0.85)		2.25 (1.01)	3.64 (0.96)		
Bachelor's degree	150 (25.1)	2.05 (0.98)		2.19 (1.04)	3.65 (0.89)		
Graduate's degree	97 (16.2)	1.96 (0.98)		2.22 (0.90)	3.72 (1.07)		
Prefer not to answer	4						

Race			*F* = 7.28^***^		*F* = 2.64		*F* = 3.31^***^
White	234 (42.8)	1.84 (0.89)		2.12 (0.91)		3.82 (0.96)	
Black or African American	126 (23.0)	1.90 (0.70)		2.04 (0.93)		3.70 (0.90)	
Asian	118 (19.7)	2.26 (0.91)		2.36 (0.93)^*^		3.31 (0.91)	
Native American or Alaskan Native; Native Hawaiian or Other Pacific Islander; and Multiracial	80 (14.5)	1.84 (0.62)		2.19 (0.86)		3.64 (1.03)	
Prefer not to answer	43						
Ethnicity							
Not Hispanic/Latino/a/e	466 (78.5)	1.94 (0.82)	*t* = −0.09	2.17 (0.87)	*t* = 0.11	3.65 (0.94)	*t* = 0.44
Hispanic/Latino/a/e	128 (21.5)	1.94 (0.93)		2.16 (1.05)		3.61 (1.05)	
Prefer not to answer	7						

History of diagnosis			*t* = 7.13^***^		*t* = 1.88		*t* = −6.11^***^
No diagnosis	384 (63.9)	2.09(0.86)		2.23 (0.92)		3.44 (0.93)	
Self and/or family diagnosis	217 (36.1)	1.62 (0.58)		2.06 (0.85)		4.00 (0.85)	

Note. MH = mental health.

* *p* < .05.

** *p* < .01.

*** *p* < .001.

**Table 2:**
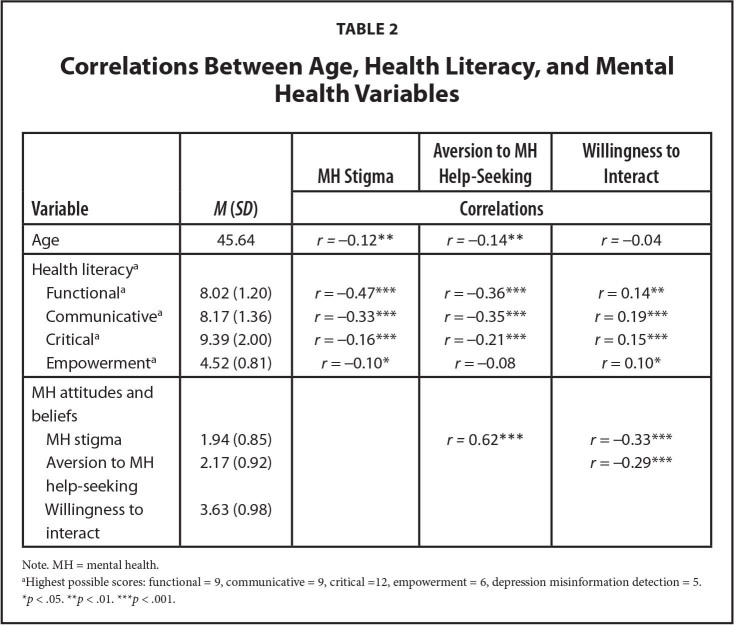
Correlations Between Age, Health Literacy, and Mental Health Variables

**Variable**	***M* (*SD*)**	**MH Stigma**	**Aversion to MH Help-Seeking**	**Willingness to Interact**

		**Correlations**		

Age	45.64	*r =* −0.12^**^	*r =* −0.14^**^	*r =* −0.04

Health literacy^a^				
Functional^a^	8.02 (1.20)	*r* = −0.47^***^	*r* = −0.36^***^	*r* = 0.14^**^
Communicative^a^	8.17 (1.36)	*r* = −0.33^***^	*r* = −0.35^***^	*r* = 0.19^***^
Critical^a^	9.39 (2.00)	*r* = −0.16^***^	*r* = −0.21^***^	*r* = 0.15^***^
Empowerment^a^	4.52 (0.81)	*r* = −0.10^*^	*r* = −0.08	*r* = 0.10^*^

MH attitudes and beliefs				
MH stigma	1.94 (0.85)		*r =* 0.62^***^	*r* = −0.33^***^
Aversion to MH help-seeking	2.17 (0.92)			*r* = −0.29^***^
Willingness to interact	3.63 (0.98)			

Note. MH = mental health.

a Highest possible scores: functional = 9, communicative = 9, critical =12, empowerment = 6, depression misinformation detection = 5.

* *p* < .05.

** *p* < .01.

*** *p* < .001.

### Relationship Between HL and MH Variables

Results of the hierarchical regressions are presented in **Table 3**. Participants who were younger, men, identified as Asian, reported no self/family history of mental illness, and scored lower on functional and communicative HL reported higher MH stigma. Participants who were younger, men, and who scored lower on functional and communicative HL reported higher aversion toward MH help-seeking. White women, individuals with a self/family history of mental illness, and those with higher communicative HL and empowerment scores were more willing to interact with individuals with mental illnesses.

**Table 3:**
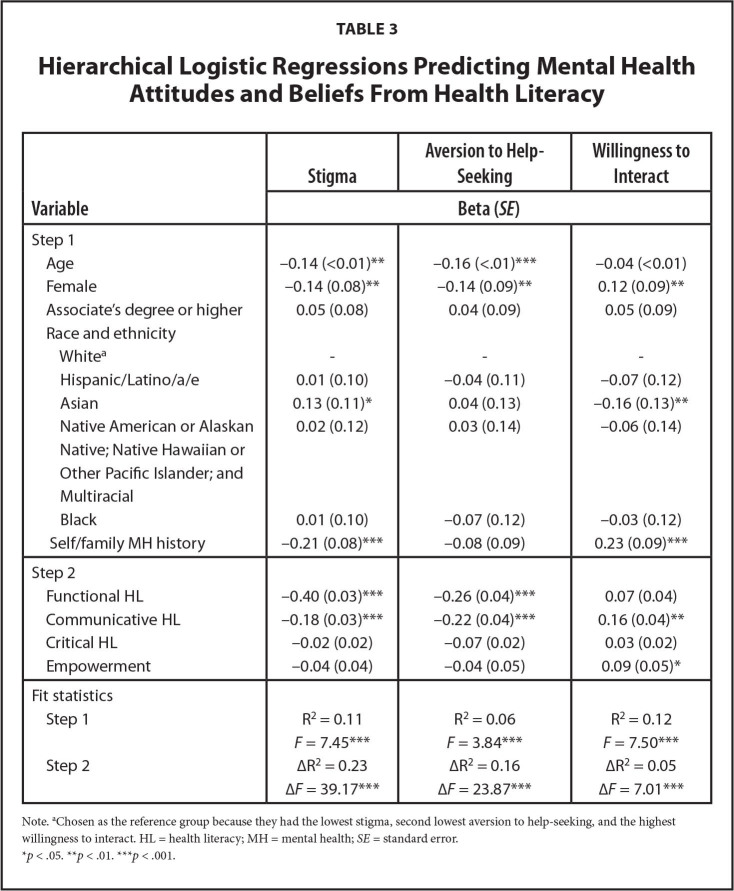
Hierarchical Logistic Regressions Predicting Mental Health Attitudes and Beliefs From Health Literacy

**Variable**	**Stigma**	**Aversion to Help-Seeking**	**Willingness to Interact**

	**Beta (*SE*)**		

Step 1			
Age	−0.14 (<0.01)^**^	−0.16 (<.01)^***^	−0.04 (<0.01)
Female	−0.14 (0.08)^**^	−0.14 (0.09)^**^	0.12 (0.09)^**^
Associate's degree or higher Race and ethnicity	0.05 (0.08)	0.04 (0.09)	0.05 (0.09)
White^a^	-	-	-
Hispanic/Latino/a/e	0.01 (0.10)	−0.04 (0.11)	−0.07 (0.12)
Asian	0.13 (0.11)^*^	0.04 (0.13)	−0.16 (0.13)^**^
Native American or Alaskan Native; Native Hawaiian or Other Pacific Islander; and Multiracial	0.02 (0.12)	0.03 (0.14)	−0.06 (0.14)
Black	0.01 (0.10)	−0.07 (0.12)	−0.03 (0.12)
Self/family MH history	−0.21 (0.08)^***^	−0.08 (0.09)	0.23 (0.09)^***^

Step 2			
Functional HL	−0.40 (0.03)***	−0.26 (0.04)^***^	0.07 (0.04)
Communicative HL	−0.18 (0.03)^***^	−0.22 (0.04)^***^	0.16 (0.04)^**^
Critical HL	−0.02 (0.02)	−0.07 (0.02)	0.03 (0.02)
Empowerment	−0.04 (0.04)	−0.04 (0.05)	0.09 (0.05)^*^

Fit statistics			
Step 1	R^2^ = 0.11	R^2^ = 0.06	R^2^ = 0.12
	*F* = 7.45^***^	*F* = 3.84^***^	*F* = 7.50^***^
Step 2	∆R^2^ = 0.23	∆R^2^ = 0.16	∆R^2^ = 0.05
	∆*F* = 39.17^***^	∆*F* = 23.87^***^	∆*F* = 7.01^***^

Note.

a Chosen as the reference group because they had the lowest stigma, second lowest aversion to help-seeking, and the highest willingness to interact. HL = health literacy; MH = mental health; *SE* = standard error.

* *p* < .05.

** *p* < .01.

*** *p* < .001.

### Direct and Indirect Effect of HL on Aversion to MH Help-Seeking

The standardized model coefficients are presented in **Table 4** (see **Table A** for unstandardized results). Significant variances were explained for both MH stigma (R^2^ = 0.31, *p* < .001) and aversion to MH help-seeking (R^2^ = 0.39, *p* < .001). Functional (beta = −0.41, *p* < .001), and communicative (beta = −0.14, *p* < .001) HL were negatively related to MH stigma. MH stigma (beta = 0.54, *p* < .001) was positively related to aversion to MH help-seeking. Communicative HL was directly (beta = −0.14, *p* = .002) and indirectly (beta = −0.09, *p* = .001) negatively related to aversion to MH help-seeking, suggesting complementary partial mediation through MH stigma (Carrión et al., 2017). Functional HL (beta = −0.22, *p* < .001) was also indirectly related to aversion to MH help-seeking through MH stigma, suggesting full mediation. Total effects were significant for functional (beta = −0.25, *p* < .001) and communicative (beta = −0.22, *p* < .001) HL.

**Table 4:**
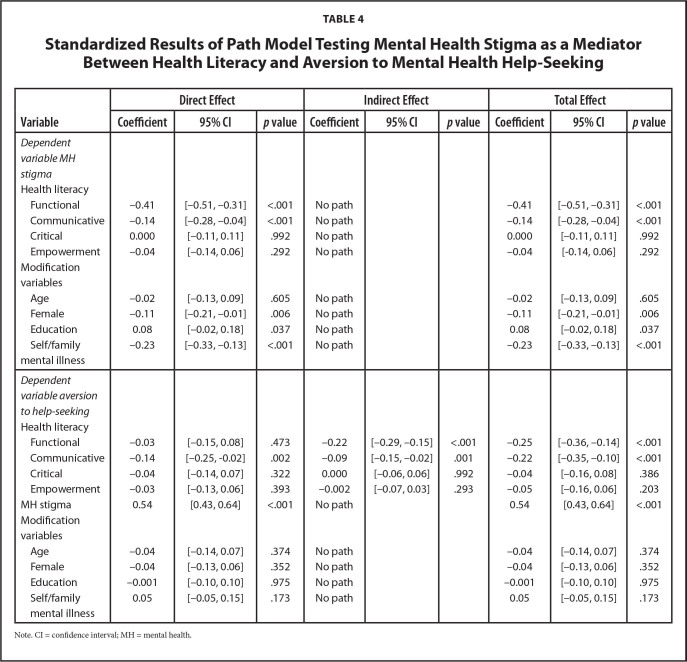
Standardized Results of Path Model Testing Mental Health Stigma as a Mediator Between Health Literacy and Aversion to Mental Health Help-Seeking

**Variable**	**Direct Effect**			**Indirect Effect**			**Total Effect**		
		
	**Coefficient**	**95% CI**	***p* value**	**Coefficient**	**95% CI**	***p* value**	**Coefficient**	**95% CI**	***p* value**

*Dependent variable MH stigma*									
Health literacy									
Functional	−0.41	[−0.51, −0.31]	<.001	No path			−0.41	[−0.51, −0.31]	<.001
Communicative	−0.14	[−0.28, −0.04]	<.001	No path			−0.14	[−0.28, −0.04]	<.001
Critical	0.000	[−0.11, 0.11]	.992	No path			0.000	[−0.11, 0.11]	.992
Empowerment	−0.04	[−0.14, 0.06]	.292	No path			−0.04	[−0.14, 0.06]	.292
Modification variables									
Age	−0.02	[−0.13, 0.09]	.605	No path			−0.02	[−0.13, 0.09]	.605
Female	−0.11	[−0.21, −0.01]	.006	No path			−0.11	[−0.21, −0.01]	.006
Education	0.08	[−0.02, 0.18]	.037	No path			0.08	[−0.02, 0.18]	.037
Self/family mental illness	−0.23	[−0.33, −0.13]	<.001	No path			−0.23	[−0.33, −0.13]	<.001

*Dependent variable aversion to help-seeking*									
Health literacy									
Functional	−0.03	[−0.15, 0.08]	.473	−0.22	[−0.29, −0.15]	<.001	−0.25	[−0.36, −0.14]	<.001
Communicative	−0.14	[−0.25, −0.02]	.002	−0.09	[−0.15, −0.02]	.001	−0.22	[−0.35, −0.10]	<.001
Critical	−0.04	[−0.14, 0.07]	.322	0.000	[−0.06, 0.06]	.992	−0.04	[−0.16, 0.08]	.386
Empowerment	−0.03	[−0.13, 0.06]	.393	−0.002	[−0.07, 0.03]	.293	−0.05	[−0.16, 0.06]	.203
MH stigma	0.54	[0.43, 0.64]	<.001	No path			0.54	[0.43, 0.64]	<.001
Modification variables									
Age	−0.04	[−0.14, 0.07]	.374	No path			−0.04	[−0.14, 0.07]	.374
Female	−0.04	[−0.13, 0.06]	.352	No path			−0.04	[−0.13, 0.06]	.352
Education	−0.001	[−0.10, 0.10]	.975	No path			−0.001	[−0.10, 0.10]	.975
Self/family mental illness	0.05	[−0.05, 0.15]	.173	No path			0.05	[−0.05, 0.15]	.173

Note. CI = confidence interval; MH = mental health.

**Table A:**
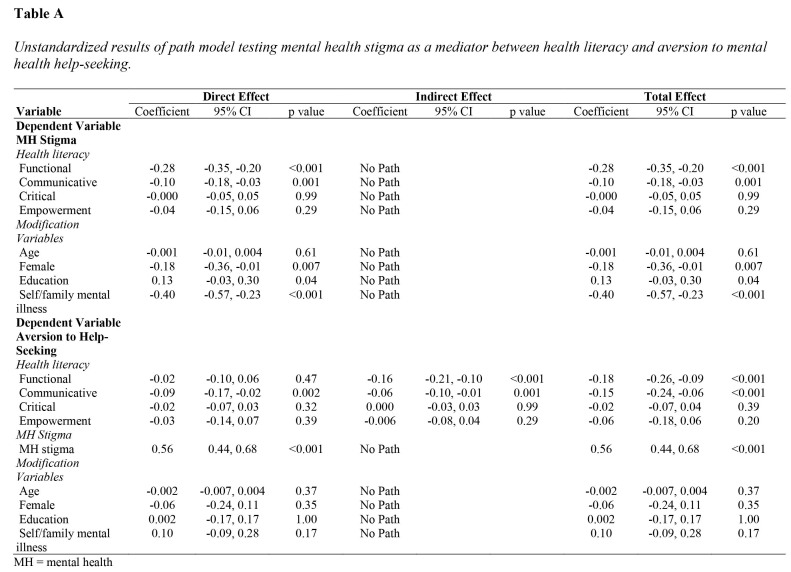
Unstandardized results of path model testing mental health stigma as a mediator between health literacy and aversion to mental health help-seeking.

**Variable**	**Direct Effect**			**Indirect Effect**			**Total Effect**		

	Coefficient	95% CI	p value	Coefficient	95% CI	p value	Coefficient	95% CI	p value
**Dependent Variable**									
**MH Stigma**									
*Health literacy*									
Functional	−0.28	−0.35, −0.20	<0.001	No Path			−0.28	−0.35, −0.20	<0.001
Communicative	−0.10	−0.18, −0.03	0.001	No Path			−0.10	−0.18, −0.03	0.001
Critical	−0.000	−0.05, 0.05	0.99	No Path			−0.000	−0.05, 0.05	0.99
Empowerment	−0.04	−0.15, 0.06	0.29	No Path			−0.04	−0.15, 0.06	0.29
*Modification*									
*Variables*									
Age	−0.001	−0.01, 0.004	0.61	No Path			−0.001	−0.01, 0.004	0.61
Female	−0.18	−0.36, −0.01	0.007	No Path			−0.18	−0.36, −0.01	0.007
Education	0.13	−0.03, 0.30	0.04	No Path			0.13	−0.03, 0.30	0.04
Self/family mental illness	−0.40	−0.57, −0.23	<0.001	No Path			−0.40	−0.57, −0.23	<0.001
**Dependent Variable Aversion to Help-Seeking**									
*Health literacy*									
Functional	−0.02	−0.10, 0.06	0.47	−0.16	−0.21, −0.10	<0.001	−0.18	−0.26, −0.09	<0.001
Communicative	−0.09	−0.17, −0.02	0.002	−0.06	−0.10, −0.01	0.001	−0.15	−0.24, −0.06	<0.001
Critical	−0.02	−0.07, 0.03	0.32	0.000	−0.03, 0.03	0.99	−0.02	−0.07, 0.04	0.39
Empowerment	−0.03	−0.14, 0.07	0.39	−0.006	−0.08, 0.04	0.29	−0.06	−0.18, 0.06	0.20
*MH Stigma*									
MH stigma	0.56	0.44, 0.68	<0.001	No Path			0.56	0.44, 0.68	<0.001
*Modification*									
*Variables*									
Age	−0.002	−0.007, 0.004	0.37	No Path			−0.002	−0.007, 0.004	0.37
Female	−0.06	−0.24, 0.11	0.35	No Path			−0.06	−0.24, 0.11	0.35
Education	0.002	−0.17, 0.17	1.00	No Path			0.002	−0.17, 0.17	1.00
Self/family mental illness	0.10	−0.09, 0.28	0.17	No Path			0.10	−0.09, 0.28	0.17

MH = mental health

### Direct and Indirect Effect of HL on Willingness to Interact with Individuals with Mental Illness

The standardized model coefficients are presented in **Table 5** (see **Table B** for unstandardized results). Significant variances were explained for both MH stigma (R^2^ = 0.32, *p* < .001) and willingness to interact (R^2^ = 0.21, *p* < .001). Functional (beta = −0.41, *p* < .001), and communicative (beta = −0.16, *p* < .001) HL were negatively related to MH stigma. MH stigma (beta = −0.30, *p* < .001) was negatively related to willingness to interact with individuals with mental illnesses. Communicative HL was directly (beta = 0.13, *p* = .014) and indirectly (beta = 0.05, *p* = .002) positively related to willingness to interact, suggesting complementary partial mediation through MH stigma. Functional HL (beta = 0.12, *p* < .001) was indirectly related to willingness to interact (via MH stigma) suggesting full mediation. The total effect was significant for communicative HL (beta = 0.17, *p* = .001). Total effect (beta = 0.05, *p* = .145) was not significant for functional HL and this effect was smaller than the indirect effect, suggesting a suppressor effect. Empowerment (beta = 0.09, *p* = .042) was positively directly related to willingness to interact with others with mental illnesses.

**Table 5:**
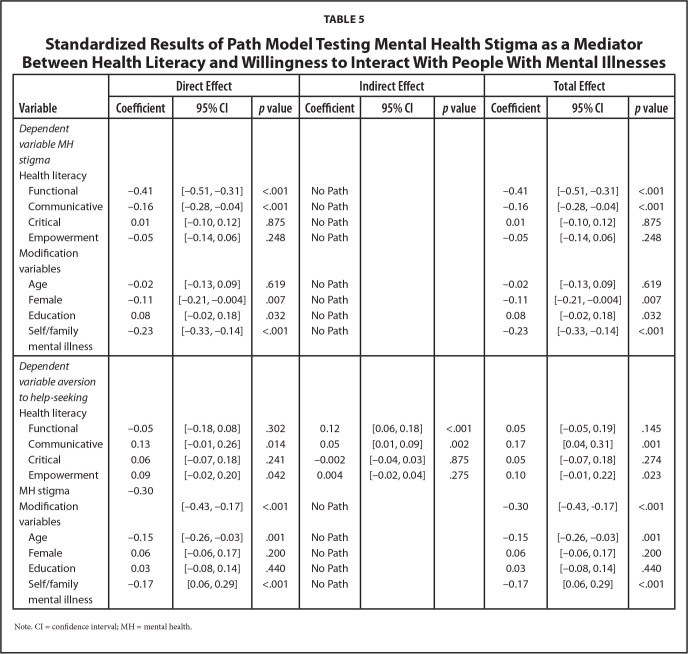
Standardized Results of Path Model Testing Mental Health Stigma as a Mediator Between Health Literacy and Willingness to Interact With People With Mental Illnesses

**Variable**	**Direct Effect**			**Indirect Effect**			**Total Effect**		
		
	**Coefficient**	**95% CI**	***p* value**	**Coefficient**	**95% CI**	***p* value**	**Coefficient**	**95% CI**	***p* value**

*Dependent variable MH stigma*									
Health literacy									
Functional	−0.41	[−0.51, −0.31]	<.001	No Path			−0.41	[−0.51, −0.31]	<.001
Communicative	−0.16	[−0.28, −0.04]	<.001	No Path			−0.16	[−0.28, −0.04]	<.001
Critical	0.01	[−0.10, 0.12]	.875	No Path			0.01	[−0.10, 0.12]	.875
Empowerment	−0.05	[−0.14, 0.06]	.248	No Path			−0.05	[−0.14, 0.06]	.248
Modification variables									
Age	−0.02	[−0.13, 0.09]	.619	No Path			−0.02	[−0.13, 0.09]	.619
Female	−0.11	[−0.21, −0.004]	.007	No Path			−0.11	[−0.21, −0.004]	.007
Education	0.08	[−0.02, 0.18]	.032	No Path			0.08	[−0.02, 0.18]	.032
Self/family mental illness	−0.23	[−0.33, −0.14]	<.001	No Path			−0.23	[−0.33, −0.14]	<.001

*Dependent variable aversion to help-seeking*									
Health literacy									
Functional	−0.05	[−0.18, 0.08]	.302	0.12	[0.06, 0.18]	<.001	0.05	[−0.05, 0.19]	.145
Communicative	0.13	[−0.01, 0.26]	.014	0.05	[0.01, 0.09]	.002	0.17	[0.04, 0.31]	.001
Critical	0.06	[−0.07, 0.18]	.241	−0.002	[−0.04, 0.03]	.875	0.05	[−0.07, 0.18]	.274
Empowerment	0.09	[−0.02, 0.20]	.042	0.004	[−0.02, 0.04]	.275	0.10	[−0.01, 0.22]	.023
MH stigma	−0.30								
Modification variables		[−0.43, −0.17]	<.001	No Path			−0.30	[−0.43, −0.17]	<.001
Age	−0.15	[−0.26, −0.03]	.001	No Path			−0.15	[−0.26, −0.03]	.001
Female	0.06	[−0.06, 0.17]	.200	No Path			0.06	[−0.06, 0.17]	.200
Education	0.03	[−0.08, 0.14]	.440	No Path			0.03	[−0.08, 0.14]	.440
Self/family mental illness	−0.17	[0.06, 0.29]	<.001	No Path			−0.17	[0.06, 0.29]	<.001

Note. CI = confidence interval; MH = mental health.

**Table B:**
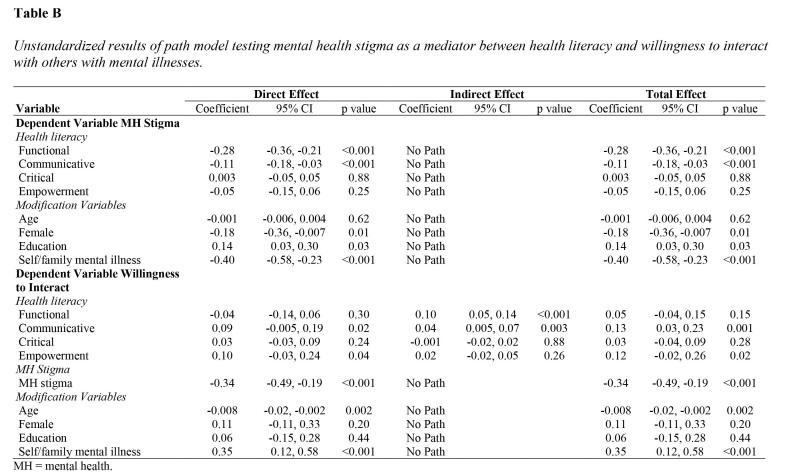
Unstandardized results of path model testing mental health stigma as a mediator between health literacy and willingness to interact with others with mental illnesses.

**Variable**	**Direct Effect**			**Indirect Effect**			**Total Effect**		

	Coefficient	95% CI	p value	Coefficient	95% CI	p value	Coefficient	95% CI	p value
**Dependent Variable MH Stigma**									
*Health literacy*									
Functional	−0.28	−0.36, −0.21	<0.001	No Path			−0.28	−0.36, −0.21	<0.001
Communicative	−0.11	−0.18, −0.03	<0.001	No Path			−0.11	−0.18, −0.03	<0.001
Critical	0.003	−0.05, 0.05	0.88	No Path			0.003	−0.05, 0.05	0.88
Empowerment	−0.05	−0.15, 0.06	0.25	No Path			−0.05	−0.15, 0.06	0.25
*Modification Variables*									
Age	−0.001	−0.006, 0.004	0.62	No Path			−0.001	−0.006, 0.004	0.62
Female	−0.18	−0.36, −0.007	0.01	No Path			−0.18	−0.36, −0.007	0.01
Education	0.14	0.03, 0.30	0.03	No Path			0.14	0.03, 0.30	0.03
Self/family mental illness	−0.40	−0.58, −0.23	<0.001	No Path			−0.40	−0.58, −0.23	<0.001
**Dependent Variable Willingness**									
**to Interact**									
*Health literacy*									
Functional	−0.04	−0.14, 0.06	0.30	0.10	0.05, 0.14	<0.001	0.05	−0.04, 0.15	0.15
Communicative	0.09	−0.005, 0.19	0.02	0.04	0.005, 0.07	0.003	0.13	0.03, 0.23	0.001
Critical	0.03	−0.03, 0.09	0.24	−0.001	−0.02, 0.02	0.88	0.03	−0.04, 0.09	0.28
Empowerment	0.10	−0.03, 0.24	0.04	0.02	−0.02, 0.05	0.26	0.12	−0.02, 0.26	0.02
*MH Stigma*									
MH stigma	−0.34	−0.49, −0.19	<0.001	No Path			−0.34	−0.49, −0.19	<0.001
*Modification Variables*									
Age	−0.008	−0.02, −0.002	0.002	No Path			−0.008	−0.02, −0.002	0.002
Female	0.11	−0.11, 0.33	0.20	No Path			0.11	−0.11, 0.33	0.20
Education	0.06	−0.15, 0.28	0.44	No Path			0.06	−0.15, 0.28	0.44
Self/family mental illness	0.35	0.12, 0.58	<0.001	No Path			0.35	0.12, 0.58	<0.001

MH = mental health.

## Discussion

This study explored the relationship between HL and MH-related attitudes and beliefs. As hypothesized, adults with higher functional and communicative HL had lower stigma and aversion to MH help-seeking. Adults with higher communicative HL and empowerment were more willing to interact with individuals with mental illnesses. MH stigma mediated the relationships between HL and aversion to MH help-seeking and willingness to interact with individuals with MH illnesses. Critical HL was not significant in any analyses. Overall, these results suggest that HL should be considered, or potentially targeted, when addressing MH attitudes.

Culturally relevant and targeted interventions may be required for groups with higher stigmatizing beliefs. In our sample, individuals with higher MH stigma and aversion to help-seeking were younger and men. Research suggests that as individuals age they become more accepting and less likely to hold stigmatizing attitudes toward those with MH problems (Bradbury, 2020). Our findings regarding gender may reflect societal norms that emphasize certain expressions of masculinity while restricting behaviors that demonstrate feelings and emotions (Chatmon, 2020) and treatment-seeking behaviors among men (Novak et al., 2019). Asian adults were also more likely to exhibit higher MH stigma and lower willingness to interact with other people with mental illnesses. This aligns with previous findings on stigma and unfavorable attitudes regarding MH in this population (Cheng et al., 2018; Jimenez et al., 2013). Conversely, adults who indicated that they and/or a family member had a history of mental illness had lower MH stigma and greater willingness to interact with others with mental illnesses, highlighting the role of personal experiences with MH in reducing stigma (Potts & Henderson, 2020). Personal experiences may expose people to realities regarding MH that counteract previously held myths or misconceptions. However, individuals may be less willing to self-disclose their mental illness due to perceived stigma from others, leading to missed opportunities for debunking stigmatizing beliefs. Therefore, interventions designed for groups with high stigmatizing beliefs and to prepare participants to provide MH first aid to those with high stigmatizing beliefs are critical to reducing stigma and improving MH help-seeking and outcomes.

Higher functional and communicative HL were associated with lower stigma and aversion to MH help-seeking. These HL skills may help individuals distinguish factual information about MH from stigmatizing and other false information. Functional HL includes skills for reading and understanding information while communicative HL includes skills for deciphering reliable information from multiple sources. These HL skills may facilitate filtering out stigmatizing information about MH often prevalent in media and cultural beliefs. Individuals with lower HL may rely on familiar information and information from trusted individuals (Chen et al., 2018; Lubetkin et al., 2015), which may be unreliable and endorse stigmatizing attitudes and aversion to help-seeking. Given the relationship between HL and stigmatizing attitudes and aversion to help-seeking, considering or including HL in MH interventions may result in improved intervention uptake and outcomes.

Communicative HL and empowerment were associated with higher willingness to interact with others. These types of HL may support engagement in fact finding that build empathy towards individuals with mental illnesses. Communicative HL may provide individuals with social and language skills and comfort to interact with individuals with mental illnesses. Additionally, given their ability to identify reliable sources and navigate multiple sources of information, those with higher communicative HL may have more accurate information about MH. Individuals with higher empowerment may have a more sophisticated understanding of MH as a product of genetic and environmental factors, rather than an individuals' shortcomings, which may promote empathy and increase their willingness to interact.

Our findings align with research on the role of stigma in MH help-seeking (Clement et al., 2015; Henderson et al., 2013; Salaheddin & Mason, 2016; Schnyder et al., 2017) and how it contributes to othering those with MH diagnoses (Augsberger et al., 2015; Mantovani et al., 2017). However, our results also highlight the importance of addressing HL to reduce MH stigma, aversion to help-seeking, and othering. As partial mediation for MH stigma was noted where communicative HL was the predictor variable, our results support addressing communicative HL to reduce aversion to help-seeking. This study is cross-sectional and the HL measures are perception-based, poor experiences with MH help-seeking and/or health systems may have impacted participants' confidence in their HL skills and intensified MH stigma and aversion to help-seeking. Similarly, negative encounters with individuals with mental illnesses may contribute to MH stigma and impact participants' confidence in engaging with others around MH. Longitudinal and qualitative studies are important for establishing the causal relationships between these variables to best inform individual-and systems-level interventions and programming.

MH first aid programs address both stigma and knowledge by providing MH education that allows individuals to accurately identify MH problems and reduce stigmatizing attitudes towards MH. To optimize MH help-seeking follow through, health care providers and other referral sources should focus on educating patients and addressing their stigmatizing attitudes while being attentive to their HL skills.

This study has several limitations. Responses to the HL and MH attitudes measures were subject to respondent biases (e.g., social desirability, inaccurate recall). Future studies should use test-based measures to assess participants' HL (e.g., Newest Vital Sign; Weiss et al., 2005) rather than perceptions-based or past behavior measures. Though the Qualtrics panel was a stratified random sample of the U.S. population, the social media sample was not representative, therefore generalizability is limited. This study should be replicated in a representative U.S. sample with sufficient subsample sizes to explore group differences. Future studies should also assess how HL effects MH attitudes longitudinally and how the uptake and outcomes of MH programs are impacted by participants' HL and including HL in the design and content of MH programs.

## Conclusions

This study assessed the relationship between HL and MH-related attitudes and beliefs. Functional and communicative HL were most implicated in MH-related attitudes and beliefs. MH stigma mediated the relationship between HL and willingness to interact with other people with mental illnesses and aversion to help-seeking. This study supports the inclusion of HL in interventions focused on MH attitudes and beliefs and reiterates the importance of addressing stigma in community and clinical settings.
